# Cutting the Chain of Discrimination during COVID-19 Pandemic by Health Literacy

**DOI:** 10.31662/jmaj.2022-0073

**Published:** 2022-09-30

**Authors:** Daiki Fujii, Maya Sophia Fujimura, Ken Ing Cherng Ong, Masamine Jimba

**Affiliations:** 1 Department of Community and Global Health, Graduate School of Medicine, The University of Tokyo, Tokyo, Japan; 2 Division of General Internal Medicine and Health Services Research, David Geffen School of Medicine, University of California, Los Angeles, USA

**Keywords:** Discrimination, COVID-19, Infodemic, Health literacy

## Abstract

The coronavirus disease 2019 (COVID-19) pandemic resulted in discrimination against patients and healthcare workers in the beginning. As more information about COVID-19 prevention became available, discrimination toward the patients and healthcare workers gradually reduced. Instead, people wearing masks in the general public were heavily discriminated when mask-wearing was recommended only for healthcare workers. After the universal use of masks was recommended, discrimination against those who were wearing masks decreased and increased among those who do not wear masks. However, due to the introduction of vaccine passports, the target for discrimination has shifted to people who have not received COVID-19 vaccines. Narrowing vaccine disparity could prevent discrimination toward unvaccinated people. However, some people are hesitating vaccination or cannot be vaccinated because of their health status. These people will remain targets for discrimination even if vaccines were equally distributed. To prevent discrimination during the COVID-19 pandemic, improving health literacy of the population could be effective in two ways. First, health literacy could reduce vaccine hesitancy by enabling people to critically evaluate vaccine information. Second, health literacy enables people to respect decisions of others to avoid vaccination. Therefore, interventions improving health literacy have the potential to contribute to cutting the chain of discrimination during the COVID-19 pandemic.

## Chain of Discrimination during the COVID-19 Pandemic

Since coronavirus disease 2019 (COVID-19) became a pandemic, discrimination related to COVID-19 has become a detrimental social issue. At the beginning of the pandemic, information and prevention activities were limited, and only minimizing human-to-human transmission was thought to prevent its spread. People feared physical contacts with those at high risks of the infection, and patients and healthcare workers became the first target for COVID-19-related discrimination ^(1)^.

As access to information on COVID-19 prevention increased, discrimination toward the patients and healthcare workers gradually decreased. Initially, the World Health Organization and Centers for Disease Control and Prevention in the United States (US) stated that wearing masks was not recommended for the general public, partially to address the sudden shortage of masks for healthcare workers. This negatively affected those with previous customs of wearing masks as a hygienic practice in some Asian countries. People wearing masks in public were heavily discriminated as if they were spreading COVID-19 into the community ^(1)^.

However, discrimination against those who were wearing masks decreased after the first asymptomatic transmission was reported. Wearing masks was considered an effective way to reduce the community transmission of COVID-19 because now people without symptoms were also a threat for transmission. Despite the lack of scientific evidence for the effectiveness of mask-wearing, many people regarded covering the mouth and nose as a logical way to prevent the transmission. The effectiveness of wearing masks was also advocated after the air transmission of COVID-19 was reported, and the universal use of masks was gradually recommended for the general public, as it was supported by scientific evidence ^(1)^. This recommendation contributed to preventing discrimination toward mask-wearing people, but increased it among people who do not wear masks.

As the pandemic progresses, the growing information about COVID-19 prevention and mask recommendation contributes to the reduction of discrimination. However, the target for discrimination has again shifted to those who have not received the COVID-19 vaccines. To overcome disease-related discrimination, various approaches have been taken, such as informational intervention and knowledge-shaping ^(2)^. In this commentary, we highlight the importance of health literacy as a promising approach to prevent discrimination during the COVID-19 pandemic.

## New Target for Discrimination: Unvaccinated People

As vaccination rates grow, some of the high-income countries have introduced vaccine passports to tighten limitations of social activities such as traveling abroad. However, vaccine passports raise an ethical question about distinguishing those who have and have not received the vaccination ^(3)^. A similar health certification was required for yellow fever in the US nearly 50 years ago. In the 19^th^ century, the immunity passport for yellow fever deprived unimmunized people of human rights such as marriage and employment ^(4)^. After half a century, history is repeating itself. Unnecessary limitations of social activities for unvaccinated people should be carefully considered in introducing vaccine passports. Although the increased uptake of the vaccines has relieved the fear of COVID-19 ^(2)^, it has brought ethical concerns for unvaccinated people ^(4)^. When introducing vaccine passports, human rights of the unvaccinated people should not be forgotten.

## Difficulties in Mitigating Discrimination toward Unvaccinated People

Equal vaccine distribution is one of the solutions in preventing discrimination toward unvaccinated people ^(4)^. Narrowing the vaccine disparity gap would contribute to both easing movement restrictions and eliminating over restrictions of social activities. However, vaccine availability is insufficient in eliminating COVID-19-related discrimination. Even when the vaccine is readily available, some people are hesitant. In 2020 in the US, 50% of Americans were uncertain or refused to be vaccinated. Effective communication will encourage people to evaluate information such as benefits and side effects critically, and it is needed for addressing the vaccine hesitancy ^(5)^.

The COVID-19 pandemic is also described as a pandemic of misinformation, or infodemic. Misinformation and distrust of vaccines are causes of the vaccine hesitancy ^(5)^. Moreover, the overabundance of disseminated information is often technical and difficult to understand. The struggle to understand the mechanism and development of the vaccine ultimately causes qualms about being vaccinated.

In contrast, some people cannot be vaccinated due to their health status. To prevent severe side effects, vaccination is not recommended for those who have a prior medical history or experience of allergies to ingredients of the vaccines. These places those at risk for becoming the targets of discrimination based on their vaccination status, especially if vaccination passports become a global mandate.

## Reducing Discrimination: What Works and What Does Not

Governments are trying to reduce the discrimination in many ways. In Japan, for example, a working group related to prejudice, discrimination, and privacy addressed the discrimination and led to law revision to punish those who discriminate patients with COVID-19 or healthcare workers ^(6)^. It regarded mass media as an enhancer of the discrimination due to excessive publicity of the patients by the local government ^(7)^. Therefore, regulating information is a possible way to reduce the discrimination.

Informational intervention is a common approach to overcoming disease-related discrimination ^(2)^. However, during the COVID-19 infodemic, constant uncertainty exists whether the information is up-to-date and valid for the future. Therefore, only providing information does not assist in overcoming COVID-19-related discrimination.

Knowledge-shaping is another approach to reduce disease-related discrimination. Higher levels of disease-related knowledge are associated with lower level of disease-related discrimination, such as the case with hepatitis B virus, HIV, and COVID-19 ^(2)^. However, due to the accumulation of scientific evidence, COVID-19 knowledge should constantly be kept updated, as it can be overturned as seen in the universal use of masks. Considering these limitations, new approaches are required to reduce COVID-19-related discrimination, in addition to enabling individuals to seek information actively and to continue updating their knowledge.

## Potential of Health Literacy to Eliminate Discrimination

Where evidence is limited or controversial, another approach may be useful to reduce discrimination. Health literacy is a potential skill for this purpose. It is defined as the ability to grasp, evaluate, and use health information effectively. In a study from Vietnam, higher levels of health literacy were associated with less fear of COVID-19 ^(8)^. Although the context is different, fear of infection of hepatitis B was positively associated with discrimination toward the patients ^(9)^. Similarly, higher levels of health literacy could be associated with less COVID-19-related discrimination. Health literacy enables people to practice risk communication, which is essential to respond to disease outbreak ^(10)^. Therefore, it was an important factor in eliminating discrimination toward mask-wearing people. Health literacy could also reduce the discrimination toward two groups of unvaccinated individuals: 1) people hesitating COVID-19 vaccine and 2) people surrounding those unvaccinated.

First, health literacy enables people who are hesitating vaccination to evaluate information about COVID-19 vaccines critically. At the beginning of the COVID-19 infodemic, people had to evaluate a plethora of flowing information, including what is misinformation, and what is reliable, evidence-based content. This struggle can be applied for vaccine information as well. If people hesitating vaccination have a high level of health literacy, they can access and evaluate information about the benefits and risks of its vaccines appropriately. Health literacy enables people who are hesitating vaccination to make informed decisions to prevent COVID-19 infection, which could encourage vaccine acceptance. As a result, health literacy could reduce the discrimination aimed at this target group.

Second, health literacy enables individuals to better understand and respect the decisions of those who avoid vaccination. Some individuals cannot be vaccinated due to pre-existing medical conditions, and others determine not to vaccinate, considering risks and benefits thoroughly. With a higher level of health literacy, empowered people can obtain accurate health-related information such as infection routes and self-protection methods. Therefore, health literacy can boost the understanding of why some people are not vaccinated and the supportive interaction with them. Therefore, improving health literacy could reduce discrimination against people who are not vaccinated as well.

## Interventions to Improve Health Literacy at the Population Level

Many interventions have been conducted to improve health literacy, but most improved only one out of three types, functional health literacy. The other two types, namely, interactive and critical health literacy, could prevent COVID-19-related discrimination through the promotion of evaluating and practicing health-seeking behaviors because they enable people to act independently and address social and economic adversity about health ^(11)^.

In a previous study to improve interactive and critical health literacy, participants joined weekly sessions to learn health behaviors such as nutrition, physical activities, and mental wellness and showed statistically significant changes in health literacy ^(11)^. New interventions are necessary to improve health literacy, which consist of providing interactive communication among their participants, such as exchanging opinions with vaccinated people about the reasons for their vaccination. This would nurture the ability to act on informed conscious decisions and reduce burden among the community. Such interventions could contribute to ending the target-shifting discrimination during the COVID-19 pandemic.

## Conclusions

Updated information about COVID-19 prevention has shifted the target for COVID-19-related discrimination, from patients and healthcare workers, to mask-wearing individuals, and to unvaccinated people. Health literacy could prevent unvaccinated people from discrimination in two ways. First, health literacy encourages people hesitating vaccination to evaluate the benefits and risks of vaccines critically, which could lead to vaccination. Second, health literacy promotes the understanding of why people avoid vaccination and initiates supportive attitudes toward them. Interactive and critical health literacy can be incorporated to promote to evaluate and practice health-seeking behaviors, which could eliminate discrimination related to COVID-19. New interventions based on these two types of health literacy could provide interactive communication among their participants, which could improve overall health literacy and contribute to ending the target-shifting discrimination during the COVID-19 pandemic.

## Article Information

### Conflicts of Interest

None

### Author Contributions

Conceptualization: DF, MJ

Data collection: DF, KICO

Writing—original draft: DF

Writing—review and editing: DF, MSF, KICO, MJ
